# The oral mucosal surface and blood vessels

**DOI:** 10.1186/1746-160X-9-8

**Published:** 2013-03-12

**Authors:** Ella A Naumova, Tobias Dierkes, Jürgen Sprang, Wolfgang H Arnold

**Affiliations:** 1 Witten/Herdecke University, Faculty of Health, School of Dentistry, Witten, Germany; 2 BWZK Koblenz, Rübenacher Straße 172, Abt.für. MKG-Chirurgie, Koblenz, 56072, Germany; 3 Praxis in der Binderstraße, Binderstraße 24, Hamburg, 20146, Germany

**Keywords:** Oral mucosa, Size, Surface, Tongue, Blood vessels

## Abstract

**Introduction:**

Detailed information about the size of the oral mucosa is scarce in the literature, and those studies that do exist do not take into account the size of the tongue or the enlargement of the surface by the papillae. Because of the various functions of the oral mucosa in the maintenance of oral health, knowledge of its true size may provide a better understanding of the physiology of the oral cavity and some oral diseases and direct future therapeutic strategies. The aim of this study was to determine the total size of the oral mucosa.

**Methods:**

Five human adult cadaver heads were cut in the median sagittal plane, and the total area of the oral surface was determined using silicon casts. The surface of the tongue was measured with quantitative profilometry. Photographs of oral blood vessels were taken in different areas of the oral mucosa of adult test subjects using intravital microscopy, and the pictures were compared with vessel casts of the oral mucosal capillaries of a maccaca fasciculrais monkey, which was studied using a scanning electron microscope.

**Results:**

The results showed that the dorsal side of the tongue comprises a large proportion of the total oral mucosal surface. The surface area of the epithelium increases moving from anterior to posterior on the tongue, and the number of underlying blood vessels increases proportionally.

**Conclusions:**

It can be concluded that the back of the tongue plays an important role in the oral resorption of drugs. Clinical relevance: The results may be of relevance for the delivery and development of oral drug application.

## Introduction

Detailed information about the size of the oral mucosa is scarce in the literature [1,2], and those studies that do exist do not take into account the size of the tongue or the enlargement of the surface by the papillae. Because of the various functions of the oral mucosa in the maintenance of oral health, knowledge of its true size may provide a better understanding of the physiology of the oral cavity and some oral diseases and direct future therapeutic strategies.

The oral surface is covered with a thin biofilm that is composed of saliva [1,3], bacteria [4] and various proteins and mucins [5]. This biofilm also serves as a reservoir for fluoride [6], and together with the oral mucosa, it plays an important role in the protection of underlying tissues. [7] shows that the surface structure of the oral epithelium is important for the defensive function of the oral mucosa. The relatively large tongue surface serves as a niche for oral bacteria and may be responsible for some types of halitosis [8]. Distribution of the oral bacteria on the surface of the mucosa varies with location and may be dependent on the surface structure of the mucosa [4]. Another function of the oral mucosa, specifically the mucosa of the tongue, is its haptic perception [9] and gustatory sensation. In this context, the surface of the tongue plays an important role because the papillae of the tongue increase the surface area and the sensitivity of the tongue.

Another important function of the oral mucosa is substance resorption. Low molecular weight molecules are transported through the oral epithelium into blood vessels. Oral mucosal drug application is an alternative to injection or enteral application [10]. The blood supply of the oral mucosa is very dense, and the architecture of the capillaries is crucial for effective drug resorption.

The aim of this study is to determine the surface area of the oral cavity quantitatively and to study the architecture of the underlying capillaries qualitatively.

## Material and Methods

Five heads of two female and three male human cadavers (age 65 – 75 years). The cadavers were provided by the Department of Anatomy II, Friedrich-Alexander-Universität Erlangen-Nürnberg and were official testamentary donations of volunteers to the Department for the anatomical student course for medical and dental students. They were fixed in 10% formalin and cut into halves along the median sagittal plane. Silicon casts were made of the oral cavities using Imprint II Garant 9371 Heavy Body® (Espe, Neuss, Germany). The surfaces of the casts were covered with aluminium foil. The foil was then glued onto paper, the outlines of the foils were digitized into AutoCAD 2011 (Autodesk GmbH, Munich, Germany) and the total surface areas were calculated in cm^2^. Because the papillae of the tongue enlarge the surface area, the casts of the surfaces of the dorsal side of the tongue were investigated with a profilometer (Infinite focus, Alicona Imaging GmbH, Graz, Austria), which determines a magnification scale of the irregular tongue surface (real surface). The surface area of the dorsal side of the tongue was divided into the following five zones: anterior third, middle third, posterior third, lateral surface and root.

Photographs of the mucosal blood vessels of the back of the tongue, the gingiva and the lips were taken on 17 healthy volunteers with an ophthalmoscope (Zeiss, Oberkochen, Germany) that was equipped with a 25 mm photo camera lens. The volunteers were between 18 and 30 years of age. Eight female and nine male subjects took part in this investigation. Selection criteria were: nonsmokers, no gingivitis or periodontitis, no oral mucosal inflammation or other mucosal diseases and no hypertension. All volunteers agreed by verbal and written consent to the oral inspection. These studies were done in 1982. By that time according to §40 German Medicines Law (Arzneimittelgesetz) this was a non invasive intervention an approval of the ethics committee was not required. The study was carried out in accordance to the Helsinki Declaration from 1964 in its revised form from 1975. The ophthalmoscopic photos of the human oral cavity were compared with the scanning electron microscope photographs of the maccaca fascicularis monkey casts.

The detailed morphology of the blood vessels was investigated using corrosion specimens of the vessels of a maccaca fascicularis. These corrosion specimens were made in 1978 in the Department of Anatomy of the University of Erlangen-Nünberg. The animal was sacrificed with an intravenous injection of pheonobarbital during a medical experiment which was approved by the University of Erlangen-Nürnberg. The cast are now in the archive of the Depatment of Anatomy of Witten/Herdecke University. The casts were obtained by perfusion of the common carotid artery with Adaldit® (Kulzer, Wehrheim, Germany), which was adjusted to the viscosity of blood using acetone. After polymerization, the soft tissues were macerated with 10% KOH. After washing with acetone, the casts were sputtered with gold-palladium (SCD 050 sputter coater, Blazers, Lichtenstein) and investigated with a Zeiss Sigma VP scanning electron microscope with 15 kV acceleration voltage using a secondary electron detector. The number of blood vessels of the back of the tongue was counted per mm^2^ in the tip, the middle and the posterior.

## Results and discussion

The surface of the dorsal side of the tongue increased continually from the anterior third towards the posterior third. In total, the mean surface area of the oral mucosa was 196.96 ± 24.20 cm^2.^ All results are summarized in Table 1. The largest area of the oral mucosa was 240.81 cm^2^; the smallest area was 153.30 cm^2^.

**Table 1:** **Quantitative measurements of surface areas of the oral mucosa in cm**^**2**^

**Oral surface**	**Mean area**	**STD**	**Median**	**Max**	**Min**
Lateral surface of the tongue	8.50	1.46	8.094	10.94	7.30
Anterior third of the dorsal side of the tongue	14.53	2.37	14.976	16.99	11.90
Middle third of the dorsal side of the tongue	26.59	3.52	28.266	28.52	20.37
Posterior third of the dorsal side of the tongue	29.86	4.40	31.824	32.55	22.05
Root of the tongue	11.37	2.65	12.16	13.80	8.12
Smooth surfaces	106.09	20.26	99.22	138.00	83.56
**Total surface**	**196.96**	**24.20**	**190.568**	**240.81**	**153.30**

In accordance with the increasing surface area from the anterior third to the posterior third of the tongue, the number of blood vessels per cm^2^ also increased. In the anterior third, 1208 ± 38 vessels per cm^2^ were counted, whereas in the posterior third, 1292 ± 46 were found. These results are summarized in Table 2.

**Table 2:** **Number of blood vessels per cm**^**2 **^**beneath the mucosal membrane of different areas of the tongue**

**Area of the tongue**	**Mean**	**STD**	**Median**	**Max**	**Min**
Lateral surface	1048	48	1050	1120	990
Anterior third	1208	38	1195	1290	1170
Middle third	1230	31	1225	1300	1180
Posterior third	1292	46	1290	1350	1240

**Figure 1:**
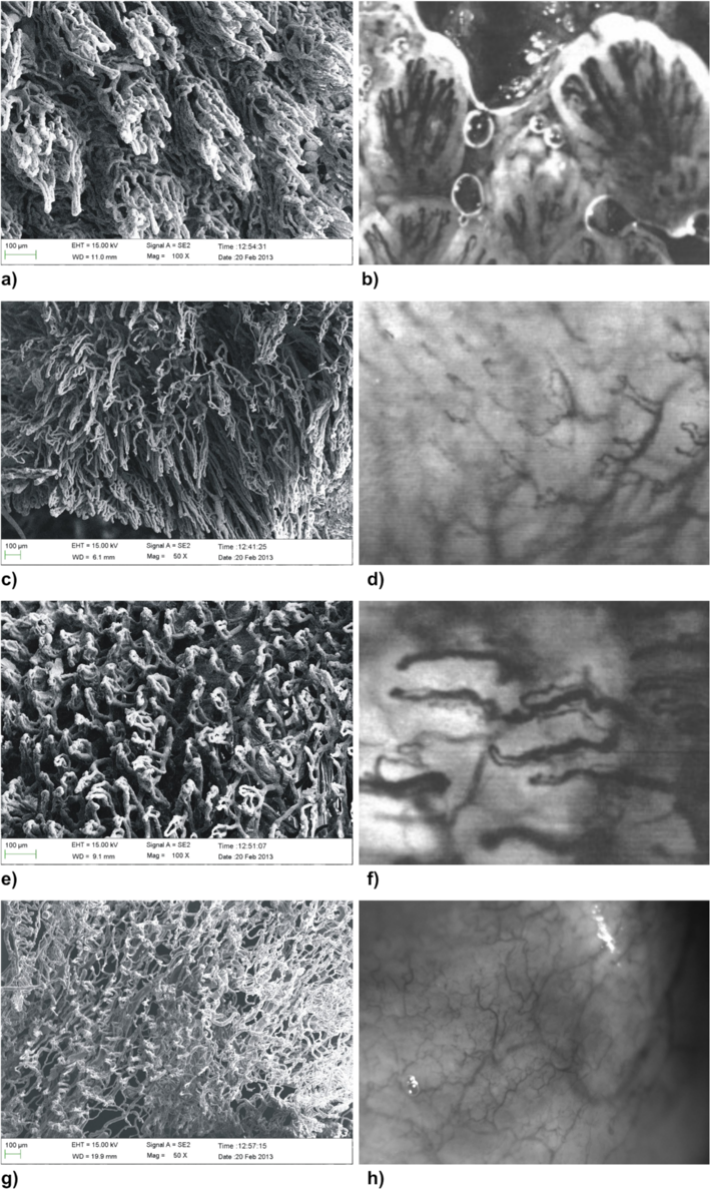
**Microphotographs of the capillary network of different areas of the oral mucosa. a**) Scanning electron micrograph of the back of the tongue, showing very dense loops of the vessels beneath the epithelium. In the middle of the picture, the vessels of a fungiform papilla can be observed (corrosion specimen of a maccaca fascicularis monkey blood vessel cast). **b**) Intravital microscopy of the vessels of human fungiform papillae in the back of the tongue, showing similar capillary loops. **c**) Scanning electron micrograph of the marginal gingival surface, showing wide loops of the vessels underneath the epithelium (corrosion specimen of a maccaca fascicularis monkey blood vessel cast). The vessel loops are relatively wide. **d**) Intravital microscopy of human gingival blood, vessels showing similar wide loops of the capillaries. **e**) Scanning electron micrograph of the capillaries in the smooth oral mucosa of the vestibulum oris, showing a capillary network beneath the epithelium oriented relatively parallel to the epithelial lining (corrosion specimen of a maccaca fascicularis monkey blood vessel cast). **f**) Intravital microscopy of human blood vessels of the oral mucosa in the smooth oral mucosa of the vestibulum oris showing capillaries running parallel to the epithelial surface. **g**) Scanning electron micrograph of the calpillaries of the bottom of the oral floor, showing a dense network of blood vessels parallel to the epithelial surface. **h**) Intravital microscopy of the blood vessels of the human oral floor showing a similar network as in the scanning picture.

Remarkable differences were found in the architecture of the oral mucosal blood vessels. In the dorsal side of the tongue, the capillaries were oriented vertically to the epithelial surfaces with separate loops within the fungiform papillae (Figure 1a and 1b). The capillary loops of the dorsal side of the tongue are very narrow and densely packed. The marginal gingiva showed a dense network of capillary loops underneath the epithelial layer (Figure 1c and 1d). These loops were wide and short, whereas in the smooth oral mucosa of the cheeks and vestibulum oris, the capillaries formed a network that was oriented parallel to the epithelial surface (Figure 1e and 1f).

Knowledge of the total area of the oral cavity is important for several reasons, but not many studies have been carried out to determine the true size of the oral mucosa [1,2]. Collins and Dawes [1] calculated the total area of the oral cavity to determine the thickness of the salivary film covering the oral mucosa. The salivary film is an important reservoir for fluoride [11], and it is needed to moisten the oral cavity; it varies in thickness depending on the total surface area, salivary secretion rate and composition [1,3,5]. However, previous studies did not take into account the total size of the tongue, which is enlarged by its numerous papillae. This, together with the fact, that the specimens of this study had no dentition may explain some differences between the results of previous studies and this study. The results of this study demonstrated that the tongue surface is a major part of the total oral mucosal surface area. A limitation of this study was the lack of dentition in the specimens. Therefore, the surface area of the teeth could not be taken into account. Other studies measured the surface area of the dentition and reported a total size of 46.1 ± 4.6 mm^2^ in adults [1], and 39.4 ± 3.9 in 14 – 19 year old subjects [2]. Therefore complete dentition would increase the surface area of the mouth considerably.

Another aspect of the function of the oral mucosa is its resorptive function. Drug delivery via the oral mucosa is becoming increasingly important because it is a comfortable method of delivery and it avoids hepatic degradation processes [10]. Transmucosal transport is dependent on the morphology of the epithelium, the underlying blood supply and the physicochemical properties of the drug. The morphology of the oral mucosa varies from keratinized squamous epithelium at the back of the tongue and parts of the gingiva and palate to non-keratinized squamous epithelium with varying thickness in the cheeks and the floor of the mouth. Thus, different areas of the oral mucosa have different repsorptive potentials and defense mechanisms [7]. The blood supply of these areas also varies. This study showed remarkable morphological differences among the different areas of the oral cavity. In the areas of non-keratinized squamous epithelium in the vestibulum oris and oral floor, the blood vessels were oriented parallel to the basal membrane of the epithelium, providing a maximal contact area with the epithelium. In the posterior tongue, the small blood vessels formed straight loops within the papillae occultae of the basal epithelial interdigitations, which resulted in a very dense capillary network. Because the epithelium of the back of the tongue is not completely keratinized, the relatively large surface area of the tongue may play an important role in oral resorption of drugs and other substances.

It can be concluded that the surface area of the oral cavity is enlarged by the back of the tongue, which may influence transmucosal transport into systemic circulation.

## Competing interests

The authors declare that they have no conflict of interest.

## Authors’ contributions

EAN: Wrote the manuscript. TD: Carried out the measurements of the surface and SEM. JS: Carried out the intravital microscopy. WHA: Was supervisor of the whole project, did manuscript corrections. All authors read and approved the final manuscript.
